# Management of Attention Deficits and Behavioral Dyscontrol With an Evening-Dosed Methylphenidate Formulation in Smith-Magenis Syndrome

**DOI:** 10.7759/cureus.21255

**Published:** 2022-01-14

**Authors:** Matthew R Narlesky, Devin McDonald, Brian Bradford, Suporn Sukpraprut-Braaten, Robert Strayhan

**Affiliations:** 1 Psychiatry, Unity Health, Searcy, USA; 2 Child and Adolescent Psychiatry, University of Utah School of Medicine, Salt Lake City, USA; 3 Research, Unity Health, Searcy, USA

**Keywords:** impulsivity, methylphenidate, melatonin agonist, behavioral dyscontrol, jornay, inverted circadian rhythm, sms, 17p11.2 deletion, microdeletion, smith-magenis syndrome

## Abstract

Smith-Magenis syndrome (SMS) is a severe neurodevelopmental disorder characterized by intellectual disability, sleep abnormalities, behavioral dyscontrol, and a distinct somatic phenotype. This report describes the case of a 10-year-old female with SMS who presented with aggression, self-injurious behavior, impulsivity, and attention deficits. She had failed trials of several stimulants and clonidine prior to presentation. An evening-dosed, delayed-release/extended-release methylphenidate formulation was added to her regimen, and she demonstrated significant improvement in her presenting symptoms. To our knowledge, this is the first published case of the use of an evening-dosed, delayed-release/extended-release methylphenidate formulation in a patient with SMS. This case highlights the need for further research on the role of these medications in managing behavioral and attentional symptoms associated with SMS.

## Introduction

Smith-Magenis syndrome (SMS) is a neurodevelopmental disorder caused by a microdeletion on chromosome 17. SMS was first documented in 1982 when Smith et al. described an association between a deletion of the chromosome 17 short arm and specific facial and cardiac abnormalities [1]. Since the initial discovery of SMS, research has characterized its genetic underpinnings, somatic dysmorphism, intellectual disability, sleep disturbance, behavioral dyscontrol, and other features. SMS typically presents during infancy due to a unique somatic phenotype and developmental delay. Several disorders with developmental delay, including Williams syndrome, Down syndrome, and brachydactyly-intellectual deficit syndrome, should be included in the differential diagnoses [2].

SMS is caused by an interstitial deletion on chromosome 17p11.2, which is typically confirmed by fluorescence in situ hybridization (FISH) [3,4]. While FISH has been the predominant method for diagnosis, new, more efficient methods, such as multiplex ligation-dependent probe amplification and real-time quantitative polymerase chain reaction, are increasingly used in the initial diagnosis [5]. The most frequent mutation occurs at 17p.11.2 *RAI1*, although mutations at 17p.11.2 *TNFRSF13B* and *MFAP4* have also been implicated [6]. The prevalence of SMS is estimated at 1 in 25,000 live births [4]. Presenting features often include intellectual disability, craniofacial abnormalities, sensorineural loss, short stature, self-injurious behavior, polyembolokoilamania, hyperactivity, inattentiveness, metabolic derangements, cardiovascular abnormalities, epilepsy, and a uniquely inverted circadian rhythm [3,7,8]. Because SMS patients are typically diagnosed early in life, education of caregivers is paramount for maximizing treatment response. Unfortunately, despite advancements in scientific understanding of the syndrome, there is insufficient data to characterize the prognosis of SMS [2].

Management of SMS typically involves a multimodal treatment approach by an interdisciplinary team. Non-pharmacological interventions include occupational therapy, physical therapy, speech therapy, feeding therapy, and individualized education programs [2]. A number of medications, including α_2_-adrenergic agonists, stimulants, antipsychotics, antidepressants, and benzodiazepines, have been used to address the behavioral dyscontrol associated with SMS. Although research has not established a first-line treatment, there is some indication that benzodiazepines account for less favorable results in addressing maladaptive behaviors [9]. Because the disrupted sleep pattern in SMS is related to an inversion of the circadian rhythm of melatonin, treatment typically aims at inhibiting melatonin secretion in the day and increasing melatonergic activity at night; examples of diurnal therapies include ultraviolet light therapy and β-adrenergic antagonists, such as acebutolol, which is thought to suppress melatonin secretion. Contrarily, ramelteon and melatonin are dosed at bedtime to restore a more typical circadian melatonin rhythm [10]. In this report, we describe the first documented case of treating attention deficits and behavioral dyscontrol in a young patient with SMS with a delayed-release/extended-release methylphenidate formulation.

## Case presentation

This case describes a 10-year-old female who was referred to the psychiatry clinic for the management of attention deficits, behavioral dyscontrol, and aggression. The patient had been diagnosed with SMS at the age of 2 years after genetic analysis at an academic center; the specific details of her genetic analysis were not available at the time of writing this report. Her past medical history was significant for allergic rhinitis, chronic constipation, chronic nausea, pes planus, and precocious puberty. She did not have a history of cardiac, renal, or other organ system defects. At the time of presentation, her appearance was notable for short stature, midface hypoplasia, and short, broad hands. Her voice was deep and mildly hoarse. Her gait was remarkable for prominent supination while ambulating. Her presenting symptoms included aggression, attention deficits, impulsivity, communication difficulties, mood dysregulation, self-injurious behavior, stereotypy, and sleep abnormalities. She tended to wake in the middle of the night and take naps in the early afternoon. Her symptoms were generally worse in the morning. She had been undergoing regular physical therapy, occupational therapy, and speech therapy. At the time of presentation, her medications were methylphenidate ER 20 mg, orally, in the morning, dexmethylphenidate 5 mg, orally, at noon and evening, and clonidine 0.4 mg orally at bedtime each night. She had been on a combination of methylphenidate and dexmethylphenidate for four years. Prior to her current medication regimen, she had failed a three-month trial of lisdexamfetamine and a one-year trial of mixed amphetamine salts. Additionally, melatonin had been tried for sleep disturbances, but it was discontinued after the patient experienced severe nightmares.

To address the patient’s aggression and behavioral dyscontrol, aripiprazole was started and titrated to 5 mg orally at bedtime each night. The remainder of the patient’s medication regimen was continued unchanged. The patient’s symptoms appeared to improve during the first two months of treatment, but she subsequently experienced moderate weight gain and recurrence of symptoms. The patient was switched to risperidone, which was titrated to 1.5 mg orally at bedtime each night. Her behavioral dyscontrol and aggression exhibited some improvement, but she continued to struggle with attention deficits, impulsivity, and disruptive behavior, particularly in the morning. To better target the timing of the patient’s symptoms, methylphenidate ER and dexmethylphenidate were discontinued, and Jornay PM® (Ironshore Pharmaceuticals Inc, Durham, NC), an evening-dosed, delayed-release/extended-release methylphenidate formulation, was started and titrated to 60 mg orally in the evening. The patient subsequently exhibited significant improvement in her attention deficits, impulsivity, and disruptive behaviors and was free of untoward effects. Her caregiver reported the patient was able to do several hours of schoolwork in one sitting without issue. At the time of writing this report, the patient’s attention deficits and behavioral dyscontrol have been stable on this medication regimen for 10 months. Sleep disturbances continue to be an issue for the patient, although we hope to address this with recently initiated tasimelteon at bedtime. The patient continues to do well in school and participate in occupational, speech, and physical therapy.

## Discussion

Our case contributes to the body of knowledge on treating impulsivity, attention deficits, and behavioral dyscontrol in SMS with a report on the effectiveness of an evening-dosed, delayed-release/extended-release methylphenidate formulation. Because of its delayed-release/extended-release drug delivery technology, Jornay PM® can be dosed in the evening and treat symptoms early the next morning [11,12]. The starting dose of Jornay PM® in patients six years and above is 20 mg orally in the evening; the dose can be increased weekly by 20 mg per day up to a maximum dose of 100 mg per day [11]. The evening dosing was particularly helpful for the patient described in this case because her most severe symptoms occurred in the morning and she had not responded to the morning administration of immediate-release stimulants. Jornay PM®’s contraindications and side effects, such as decreased appetite, irritability, and trouble sleeping, are similar to other methylphenidate formulations [11]. Given the coexistence of irregular sleep patterns, attention deficits, and behavioral dyscontrol in SMS, further research should explore the value of evening-dosed, delayed-release/extended-release medications in treating SMS patients, particularly in cases with prominent morning symptoms.

This case highlights the need for increased clinical awareness of SMS. As SMS patients typically exhibit signs during infancy, increased awareness of SMS would better equip primary care providers to initiate early intervention [13]. Early indicators in SMS, including hypotonia, lethargy, and sleep disturbances, are typically conspicuous to the point that basic clinical awareness would warrant suspicion in providers treating undiagnosed SMS patients [13,14]. In regions that lack access to specialist care, telemedicine may prove to be a feasible consideration as video analysis has demonstrated benefit in other neurodevelopmental disorders [15,16]. Increased clinical awareness would also allow for earlier implementation of behavioral interventions and education strategies, which some have argued should take precedence over drug therapy [17,18].

Finally, our case report underscores the need for further research on pharmacologic approaches to treating SMS. The sleep disturbance associated with SMS is an auspicious target for intervention because of the dramatic impact on the patient’s daily functioning. Although clinicians have attempted to address the sleep disturbances with the diurnal administration of melatonin-suppressing β-adrenergic antagonists and nocturnal administration of melatonin, much remains to be understood about the optimal dosing and timing of the administration of these medications [10]. Additionally, the benefit of melatonin agonists, namely, ramelteon and tasimelteon, which have received FDA approval for treating nighttime sleep disturbance in SMS, is not well-characterized in the literature. Similarly, the role of medications in addressing behavioral dyscontrol is poorly understood. Studies on the use of antipsychotics in patients with SMS have yielded mixed results [17,9]. Available data on treatment with other classes of medications, including antidepressants, mood stabilizers, and α2-adrenergic agonists, is equally inconclusive [9]. Fortunately, novel approaches to treating SMS symptoms have been encouraging. Momosaki et al. reported an improvement in spasms and sleep disturbance associated with SMS after treatment with adrenocorticotropic hormone therapy, which was hypothesized to normalize the hypothalamic-pituitary-adrenal axis through the regulation of corticotropin-releasing hormone [19]. Further research on the pharmacological treatment of SMS symptoms should delineate guidelines for currently used medications and evaluate the potential of novel treatment options.

## Conclusions

SMS is an uncommon neurodevelopmental disorder associated with intellectual disability, sleep abnormalities, and behavioral dyscontrol, among other symptoms. Current pharmacologic approaches to treating attention deficits and behavioral dyscontrol, such as antipsychotics and stimulants, have yielded mixed results. Our case describes the use of an evening-dosed methylphenidate formulation in treating attention deficits, impulsivity, and behavioral dyscontrol in a patient with SMS. Because of the disrupted circadian rhythm of SMS patients, the timing of medication release is paramount to treating the symptoms. The current literature presents a basic framework for managing the disorder, but there is much to be learned about the pharmacologic treatment of the symptoms associated with SMS.
